# The Dynamic Ion Motive Force Powering the Bacterial Flagellar Motor

**DOI:** 10.3389/fmicb.2021.659464

**Published:** 2021-04-13

**Authors:** Anaïs Biquet-Bisquert, Gilles Labesse, Francesco Pedaci, Ashley L. Nord

**Affiliations:** Centre de Biologie Structurale (CBS), INSERM, CNRS, Université Montpellier, Montpellier, France

**Keywords:** ion motive force, bacterial flagellar motor, stator, bacterial electrophysiology, subunit exchange, fluctuations, ion specificity, cell-to-cell signaling

## Abstract

The bacterial flagellar motor (BFM) is a rotary molecular motor embedded in the cell membrane of numerous bacteria. It turns a flagellum which acts as a propeller, enabling bacterial motility and chemotaxis. The BFM is rotated by stator units, inner membrane protein complexes that stochastically associate to and dissociate from individual motors at a rate which depends on the mechanical and electrochemical environment. Stator units consume the ion motive force (IMF), the electrochemical gradient across the inner membrane that results from cellular respiration, converting the electrochemical energy of translocated ions into mechanical energy, imparted to the rotor. Here, we review some of the main results that form the base of our current understanding of the relationship between the IMF and the functioning of the flagellar motor. We examine a series of studies that establish a linear proportionality between IMF and motor speed, and we discuss more recent evidence that the stator units sense the IMF, altering their rates of dynamic assembly. This, in turn, raises the question of to what degree the classical dependence of motor speed on IMF is due to stator dynamics vs. the rate of ion flow through the stators. Finally, while long assumed to be static and homogeneous, there is mounting evidence that the IMF is dynamic, and that its fluctuations control important phenomena such as cell-to-cell signaling and mechanotransduction. Within the growing toolbox of single cell bacterial electrophysiology, one of the best tools to probe IMF fluctuations may, ironically, be the motor that consumes it. Perfecting our incomplete understanding of how the BFM employs the energy of ion flow will help decipher the dynamical behavior of the bacterial IMF.

## Introduction

Several bacteria propel themselves by rotating their flagella (Berg and Anderson, 1973). The bacterial flagellar motor (BFM) is the powerful molecular nanomachine at the base of each flagellum responsible for such rotation. One of the few known examples of biological rotatory machines, the BFM is unique in its remarkable power and efficiency in converting free energy into mechanical work. Flagellar rotation is driven by the ion-motive force (IMF), the electro-chemical potential difference built across the membrane during cellular respiration. The demonstration that the motor is driven not by the energy of ATP hydrolysis but by the flux of ions across the membrane (Larsen et al., 1974; Manson et al., 1977) came a few years after the realization that flagella rotate (Berg and Anderson, 1973). Given an energy source that is a charged quantized particle, moving along an electric field, both the experimental and theoretical treatments of the BFM energization are challenging. For example, under physiological conditions, the energy provided by the translocation of a single ion is about three times smaller than that released by ATP hydrolysis. As a consequence, important quantities like the elementary step size and mechanisms like the force generation are difficult to resolve experimentally and model theoretically. Ions move according to both electrical and concentration gradients. The IMF is defined as the sum of these two contributions by

(1) $$\begin{array}{l} \text{IMF}=\Delta \text{Ψ}-2.3\frac{RT}{F}\Delta pI \end{array}$$

where ΔΨ = Ψ_*int*_−Ψ_*ext*_ is the difference in membrane potential Ψ, built up by the ensemble of charges separated by the membrane, $\Delta pI=pI_{int}-pI_{ext}=\log\frac{{\left[I\right]}_{ext}}{{\left[I\right]}_{int}}$ is the difference in the specific ion (*I*) potential, [*I*] is the ion concentration, *R* is the gas constant, *F* is the Faraday constant, and *T* is the temperature (2.3*RT*/*F*≃60 *m*V at *T* = 300*K*). The IMF is maintained out of equilibrium in respiring cells by transmembrane (TM) complexes that actively pump ions outward across the inner membrane. External ions can diffuse and finally fall along the energy potential to be consumed by the BFM and by other TM complexes such as the ATP synthase.

**Figure 1:**
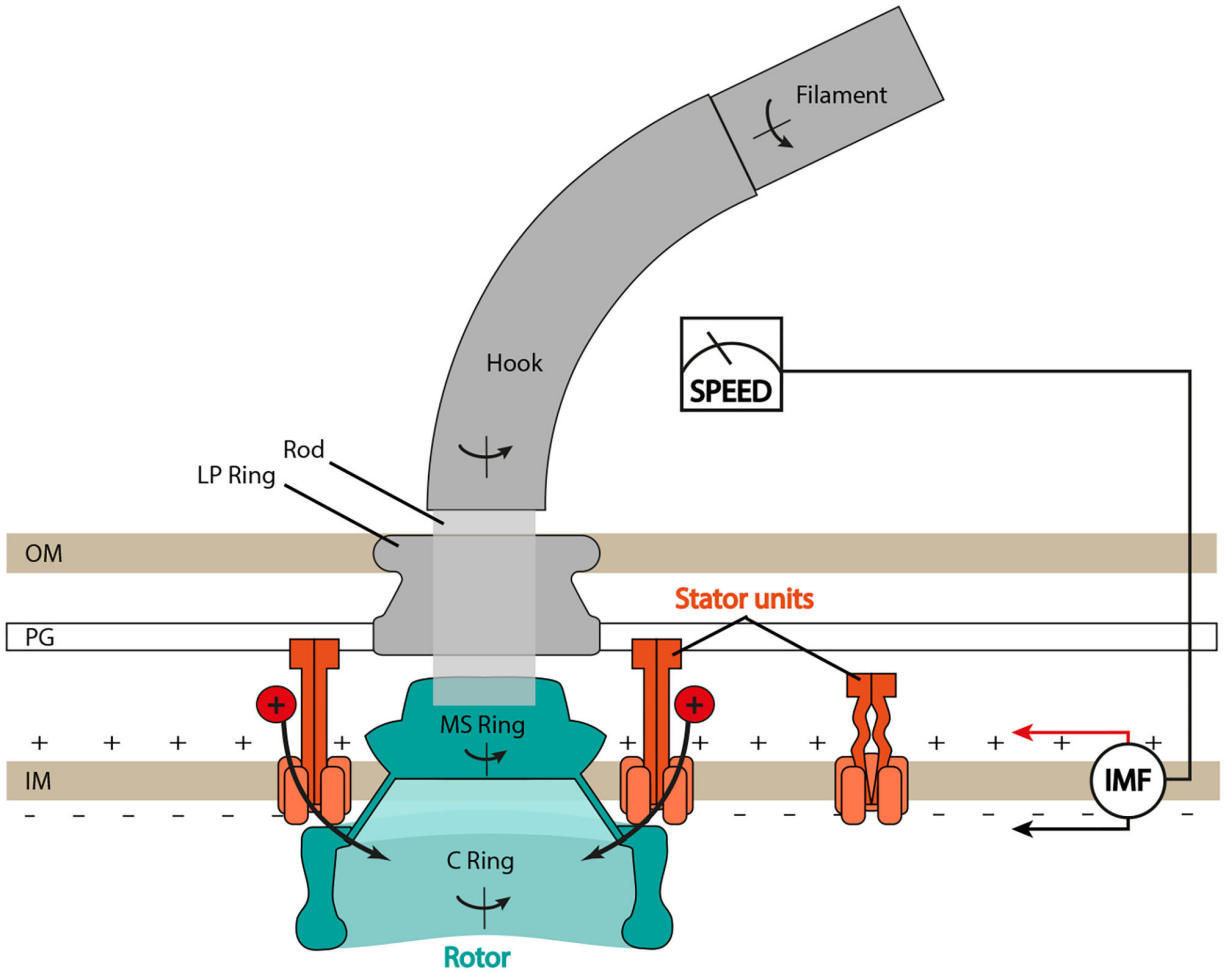
Schematic of the bacterial flagellar motor (BFM) in Gram-negative bacteria. The flagellar filament is connected to the rotor (in blue) through the hook, the universal joint, and the rod, the central driveshaft that connects the hook to the MS ring. The LP ring is assembled around the rod and acts as a bushing. Stator units bind to the peptidoglycan (PG) layer, translocate ions from the periplasm to the cytoplasm, and generate torque by interacting with the C ring. IM, inner membrane; OM, outer membrane. Type III export apparatus not shown.

Despite differences in ion selectivity and a rich diversity of evolved structural details (Rossmann and Beeby, 2018), the core structure of the BFM (see Figure 1) is well conserved. In *Escherichia coli*, the rotor is about 45 nm in diameter and composed of two main rings, the inner membrane embedded MS-ring and the cytoplasmic C-ring. The rotor couples rotation to the flagellum via the rod and then the extracellular hook. The stator consists of multiple units anchored to the peptidoglycan (PG) at the rotor's periphery. Each stator unit acts as an ion channel through which ions translocate and transfer their energy to generate the force, which rotates the rotor. Ion consumption of the stator unit is specific: MotAB stator units (e.g., in *E. coli* or *Salmonella enterica*) consume the cellular proton motive force (PMF), whereas PomAB stators (e.g., in *Vibrio alginolyticus*) consume the sodium ion motive force (SMF) Sowa and Berry (2008). Importantly, at least in enteric bacteria such as *E. coli* and *S. enterica*, stator units are not always bound to the motor complex, but they are observed to dynamically exchange between an inactive unbound state, diffusing in the inner membrane, and a motor-bound active state (Leake et al., 2006). Moreover, such exchange is mechanosensitive, as the motor can adapt the number of bound stator units depending on the external viscous load, incorporating up to a dozen (Lele et al., 2013; Tipping M. J. et al., 2013; Chawla et al., 2017; Nord et al., 2017; Terahara et al., 2017). Several studies suggest that stators may also be capable of sensing IMF, and that stator assembly is dependent upon the driving ion (Fukuoka et al., 2009; Tipping J. M. et al., 2013).

In this review, we present and discuss some of the major results that shed light on the dynamic bacterial IMF, in particular with respect to the structure and activity of the flagellar motor and its stator units.

## BFM Speed Is Proportional to IMF

Understanding the relations that couple IMF, ΔΨ, Δ*pI*, and motor activity (measured via the swimming speed of cells or the angular speed and torque of individual motors) is of great interest, particularly as it provides accurate constraints for physical mechanistic models of the motor. Unfortunately, drawing a conclusive general scheme on how the motor activity depends on the IMF components is complicated by the dependence of membrane and ion potentials on intrinsic cellular homeostasis, large cell-to-cell variability, and differences among strains. Measuring the activity of single motors under controlled conditions helps to obtain the full distribution of behaviors.

**Figure 2:**
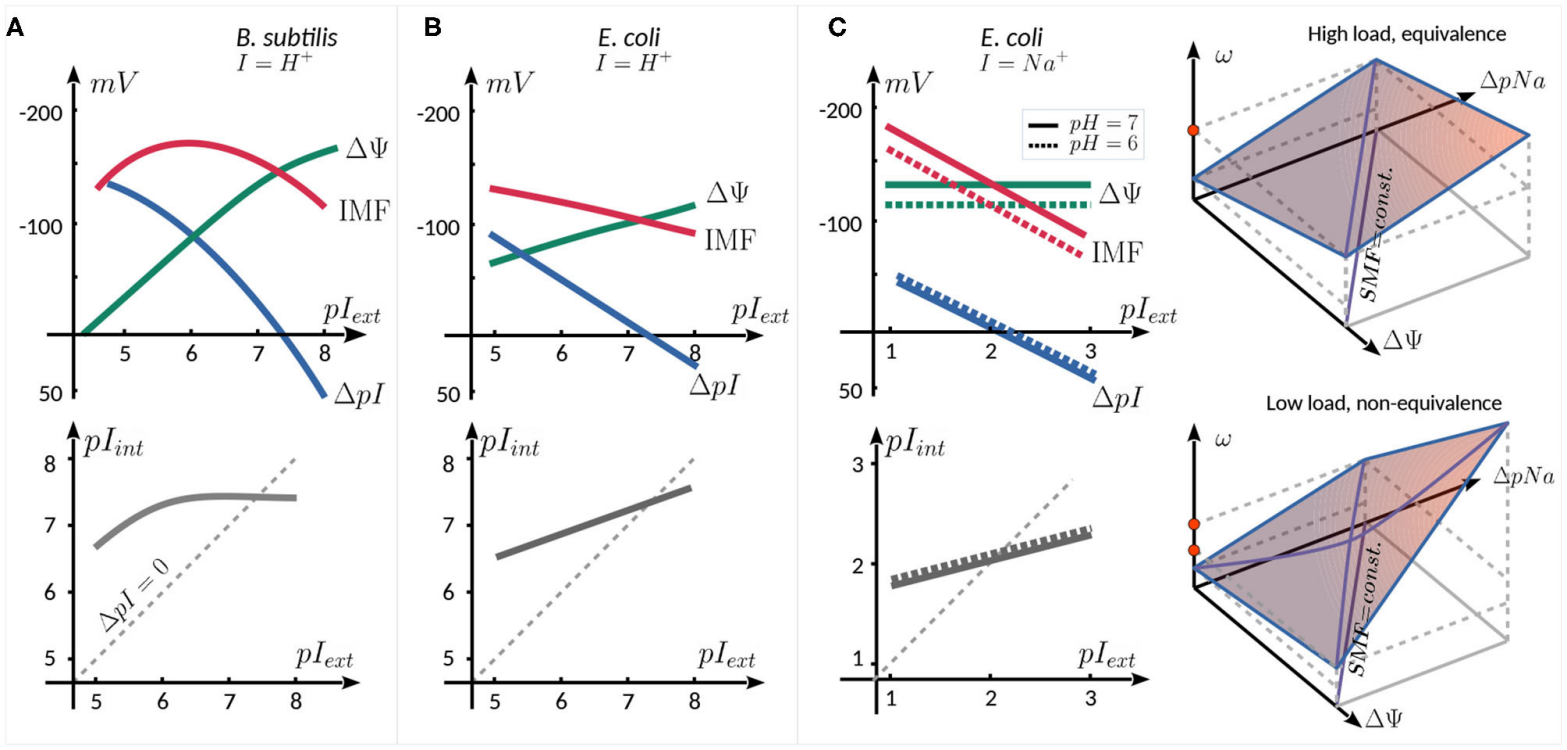
Schematic relation between ΔΨ, Δ*pI*, IMF (top panels), and *pI*_*int*_ (bottom panels) as a function of external ion concentration. The graphs are reproduced from the data published in **(A)** (Shioi et al., 1980), for the proton consuming motor of *B. subtilis*, **(B)** Minamino et al. (2003), for the proton consuming motor of *E. coli*, and **(C)** Lo et al. (2007) for the sodium consuming chimeric motor of *E. coli* employing chimeric Na^+^ stators. The two rightmost panels in **(C)** show schematically the equivalence of the SMF components at high load (top), and their nonequivalence (bottom panel, see text).

A natural parameter to vary when studying the motor behavior as a function of IMF is the concentration of the coupling ion in the external medium, affecting *pI*_*ext*_, as illustrated in Figure 2 for three published works. For proton-consuming motors (e.g., *E. coli, B. subtilis, Streptococcus*), changing *pH*_*ext*_ affects not only motor activity, but also several other cellular mechanisms. This results in a coupling between the components of the PMF. In *B. subtilis* (Figure 2A) (Shioi et al., 1980) and *E. coli* (Figure 2B) (Minamino et al., 2003), an increase in *pH*_*ext*_ is accompanied by an increase in *pH*_*int*_ (linear in *E. coli*, with a saturation in *B. subtilis*), with *pH*_*int*_ > *pH*_*ext*_ up to *pH*_*ext*_ ~ 7.5. As Δ*pH* decreases, ΔΨ increases in a compensatory manner, resulting in a PMF that does not change dramatically (within ~ 50 mV) in the range of *pH*_*ext*_ = 5−8. The coupling between *pI*_*ext*_ and ΔΨ has been cleverly avoided by expressing chimeric Na^+^-driven stators in *E. coli* (Lo et al., 2007) (Figure 2C). In this case, varying *pNa*_*ext*_ has very little effect on ΔΨ. Therefore, the SMF follows Δ*pNa*, decreasing with increasing *pNa*_*ext*_, given that *pNa*_*int*_ increases less rapidly than *pNa*_*ext*_. Moreover, *pH*_*ext*_ can also be used to control the SMF, as it linearly affects ΔΨ but is almost entirely decoupled from Δ*pNa*.

In the Na^+^-driven chimeric motor of *E. coli*, at high load the speed of the motor is linearly proportional to SMF, with an equivalent dependence upon ΔΨ (controlled by *pH*_*ext*_) and Δ*pNa* (controlled by *pNa*_*ext*_). At lower load (0.35 μm beads), the equivalence between the two components breaks down (Figure 2C, right panels). For instance, if ΔΨ is decreased and Δ*pNa* is increased so as to keep the SMF constant, one observes an increase in motor speed. The non-equivalence is graphically shown in the space (Δψ, Δ*pNa*, ω) in Figure 2C: : while at high load, the same speed (red dot) can be reached by increasing either Δψ or Δ*pNa*, at low load, this is no longer true (red dots), as the slope of ω, seen as a function of one component, depends on the other component. The leading hypothesis to explain this non-equivalence is that the rate-limiting step at low load and low [Na^+^]_*ext*_ conditions is the diffusion-limited binding of Na^+^ ions to the stator units (Lo et al., 2007).

Despite some descriptions of nonlinearities (threshold, saturation, and the non-equivalence of the IMF components; Khan and Macnab, 1980; Shioi et al., 1980; Lo et al., 2007), reflecting the complexity of the measurements and of their interpretation, numerous results contribute to a general consensus that there exists a linear relationship between the speed of the motor and IMF. Table 1 summarizes some of the published works that have described this proportionality and the range of IMF for which it holds, covering the physiological range. In Lo et al. (2007), the authors investigated motor speed per stator unit in Na^+^-powered motors, finding that the linear relationship between speed and IMF holds true at the level of an individual stator unit. However, the proportionality breaks down with acidification of the cytoplasm, at least in H^+^-powered motors. In the presence of a weak acid (which dissociates upon crossing the cytoplasmic membrane, lowering *pH*_*int*_ and partially collapsing Δ*pH*), a decrease of *pH*_*ext*_ from 7 to 5 leads to a total decrease in PMF of only ~ 10 mV, yet causes a strong decline in motor speed. From these results, it was hypothesized that high internal proton concentration hinders proton unbinding from the stators in the cytosol, hampering their function (Minamino et al., 2003). Therefore, the motor seem to respond to the entire IMF, unless *pH*_*int*_ is lowered substantially (Gabel and Berg, 2003).

**Table 1:** Published linearity ranges between motor speed ω and ion motive force (IMF) in different strains.

**Strain**	**Ion**	**IMF (mV)**	**ω (Hz)**	**Load**	**Comment**	**References**
*B. subtilis*	H^+^	−30, −100		H	Threshold, saturation	Shioi et al., 1980
*Streptococcus*	H^+^	−30, −100	0.5, 2	H		Manson et al., 1980
*Streptococcus*	H^+^	0, −50	0, 4	H		Khan et al., 1985
*Streptococcus*	H^+^		20, 70	H	H^+^ flux linear with ω	Meister et al., 1987
*Streptococcus*	H^+^	20, −200	0, 10	H		Khan et al., 1990
*E. coli*	H^+^	−50, −150	0.1, 7	H	Voltage via micropipette	Fung and Berg, 1995
*E. coli*	H^+^	0, −150^*a*^	0, 5	H	Two motors of one cell	Gabel and Berg, 2003
*E. coli*	H^+^	0, −150^*a*^	10, 250	L	Two motors of one cell	Gabel and Berg, 2003
*V. alginolyticus*	Na^+^	−130, −180^*b*^	50, 700	H/L	Torque-speed curves	Sowa et al., 2003
*E. coli*	H^+^	−100, −130		H	Const. swimming speed	Minamino et al., 2003
*E. coli*	Na^+^	−54, −187	2.2, 8.7^*c*^	H	pH_*ext*_ [Na^+^]_*ext*_ controlled	Lo et al., 2007

*The IMF column indicates the range tested. The load column indicates high (H) (tethered cell or ~ 1μm bead assays) or low (L) load (350–450 nm bead assay) applied to the motor*.

a *Assumed*.

b *Calculated from Lo et al. (2007)*.

c *Speed of first stator*.

When considering the linearity between IMF and speed, one should also keep in mind the associated time scale. Because of technical limitations, mainly in the dynamical measurement of IMF, the linear relationship has been experimentally demonstrated only at low frequency, i.e., in ensemble averages of ω and IMF acquired over relatively long periods of time. While it is now possible to measure ω with ~ 10 ms resolution in bead assays, the IMF is often measured on a scale of several seconds to minutes, and often averaged over the entire cell population. However, at least two assays have managed to directly and dynamically manipulate the IMF. By applying a varying external voltage directly to a trapped and permeabilized cell within a micropipette, the linearity between ω and IMF was demonstrated with a resolution of a few seconds (Fung and Berg, 1995). By expressing and exciting the proton pump proteorhodopsin to trigger proton efflux (Walter et al., 2007; Tipping J. M. et al., 2013), the IMF could be dynamically perturbed (though not measured) while measuring ω with sub-second resolution. Below, we discuss recent techniques for dynamic IMF quantification. When observing motor speed and IMF at higher frequency (i.e., averaging on sub-second time intervals), the analysis is complicated by fluctuations, and resolving the relationship between fast IMF fluctuations and fast speed variations remains challenging. Finally, as discussed further below, dynamic stator assembly and exchange must also be considered (or controlled) when studying the relation between IMF and speed.

The observation that the ion flow through the motor is directly proportional to speed, together with a linear relation between IMF and speed, contributed to a widely used model wherein ion translocation is tightly coupled to rotation, i.e., a fixed number of ions yields a fixed angular displacement (Manson et al., 1980; Meister et al., 1987). The IMF-speed linearity is a necessary condition for the tight-coupling model, and it has been validated by several measurements, as described above. However, it is not a sufficient condition. Given its importance for BFM mechanistic modeling, as well as other microbiological mechanisms (e.g., mechanosensing and biofilm initiation; Belas, 2013), the tight-coupling hypothesis warrants continued scrutiny, ideally at the single motor level.

## Stator Assembly Is Sensitive to IMF

Contrary to the long-held assumption that, once assembled, the structure of the BFM was static, it is now well established that multiple motor components dynamically exchange. The most well-studied example of this are the stator units, which exchange between an active motor-bound form and an inactive membrane diffusing form on a timescale of seconds to minutes (Leake et al., 2006). The rate of exchange and the steady-state number of stators is dependent upon the viscous load on the motor (Lele et al., 2013; Tipping M. J. et al., 2013; Chawla et al., 2017; Nord et al., 2017), a property that allows the bacterium to adapt to changes in local viscosity. However, there are multiple clues that suggest that stator exchange and assembly is sensitive not only to the local mechanical environment, but also to the local electrochemical environment. Such a capacity would allow strains that couple multiple ions via separate stators to tune their motor composition to the prevailing conditions (Thormann and Paulick, 2010).

One of the first experiments to suggest that stator assembly was sensitive to IMF was Fung and Berg's micropipette experiment (Fung and Berg, 1995). Once a filamentous cell of *E. coli* was installed in the micropipette, they dissipated the PMF via an ionophore, which halted motor rotation, as expected. But, when the membrane voltage was reinstated, they observed a delay before motors began rotating, after which speed increased in step-wise increments. This suggested that stator units inactivated, in some way, upon PMF collapse, then individually reactivated or reassembled upon reinstatement of the PMF. In an attempt to hold the number of stator units constant while assessing the linearity between speed and IMF, the authors avoided total PMF collapse by maintaining a nonzero DC voltage. However, IMF-dependent stator association was less evident in later experiments of the activity of two motors on the same *E. coli* cell during slow collapse of the IMF. The two motors reached zero speed at the same time, potentially suggesting that the stator units did not dissociate or inactivate at vanishing IMF (Gabel and Berg, 2003), though the low temporal resolution (~ 5 s) leaves room for uncertainty.

Tipping J. M. et al. (2013) used fluorescent protein (FP)-tagged stators to show that, upon collapse of the PMF in *E. coli*, stators dissociate from the motor on a timescale of minutes. However, these results are in disagreement with two separate studies that, also using FP-tagged stators, observe that the PMF is not necessary for stator assembly in either *S. enterica* or *E. coli* (Morimoto et al., 2010; Suzuki et al., 2019). While it seems well established that the ΔpH component of the PMF is not necessary for assembly in *E. coli* or *S. enterica* (Fung and Berg, 1995; Morimoto et al., 2010; Suzuki et al., 2019), recent work has observed that assembly increases at lower external pH (Suzuki et al., 2019). Thus, the concentration of accessible protons seems to be more important than the gradient across the membrane. Yet, surprisingly, mutation of a conserved protonatable residue in MotB, which renders the stator nonfunctional, has shown that proton translocation is not necessary for stator assembly in *E. coli* or *S. enterica* (Zhou et al., 1998; Kojima and Blair, 2001; Morimoto et al., 2010; Suzuki et al., 2019).

For the Na^+^ stator, SMF is required for assembly in *V. alginolyticus* (Fukuoka et al., 2009), and contrary to the H^+^ stator, Na^+^ conduction is also required for assembly in *V. alginolyticus* and *B. subtilis* (Fukuoka et al., 2009; Terahara et al., 2017). And while the concentration of external Na^+^ is crucial for stator assembly in *V. alginolyticus, B. subtilis*, and the Na^+^-driven hybrid stator in *E. coli* (Fukuoka et al., 2009; Sowa et al., 2014; Terahara et al., 2017), it is not necessary for Na^+^-stator assembly in *S. oneidensis* (Paulick et al., 2015).

We summarize the studies to date which probe the ion-related conditions necessary for stator assembly in Table 2. There is not yet a coherent universal picture of stator ion sensing; as with other behaviors, it may prove ion and species dependent, though the external concentration of coupling ion seems generally important. It remains to be shown whether this is due to an ion sensing capability of the stator. Alternatively, if stator binding to the PG is stabilized by force (Chawla et al., 2017; Nord et al., 2017), it may be simply the case that increasing ion concentration and rate of ion conduction increases, on average, the stability of the stator-PG bond. The fact that ion conduction in not required for H^+^ stator assembly argues against this explanation in the case of the H^+^-stator.

**Table 2:** Ion motive force (IMF) and ion related conditions shown to be necessary, ✓, or not, ✗, for stator assembly.

**Condition**	**Necessary for assembly?**	**Ion**	**Species**	**References**
PMF	(✓)	H^+^	*E. coli*	Fung and Berg, 1995
	✓	H^+^	*E. coli*	Tipping J. M. et al., 2013
	✗	H^+^	*E. coli*	Suzuki et al., 2019
	✗	H^+^	*S. enterica*	Morimoto et al., 2010
	✗	H^+^	*S. enterica*	Suzuki et al., 2019
SMF	✓	Na^+^	*V. alginolyticus*	Fukuoka et al., 2009
ΔpH	✗	H^+^	*E. coli*	Fung and Berg, 1995
	✗	H^+^	*E. coli*	Suzuki et al., 2019
	✗	H^+^	*S. enterica*	Morimoto et al., 2010
	✗	H^+^	*S. enterica*	Suzuki et al., 2019
pH_ext_	✓	H^+^	*E. coli*	Suzuki et al., 2019
	✓	H^+^	*S. enterica*	Suzuki et al., 2019
[Na^+^]_ext_ or ΔpNa	(✓)	Na^+^	*E. coli*	Sowa et al., 2005
	(✓)	Na^+^	*E. coli*	Sowa et al., 2014
	✓	Na^+^	*V. alginolyticus*	Fukuoka et al., 2009
	✓	Na^+^	*B. subtilis*	Terahara et al., 2017
	✗	Na^+^	*S. oneidensis*	Paulick et al., 2015
	✗	H^+^	*S. oneidensis*	Paulick et al., 2009
	✗	H^+^	*B. subtilis*	Terahara et al., 2017
H^+^ conduction	(✗)	H^+^	*E. coli*	Zhou et al., 1998
	(✗)	H^+^	*E. coli*	Kojima and Blair, 2001
	✗	H^+^	*E. coli*	Suzuki et al., 2019
	✗	H^+^	*S. enterica*	Morimoto et al., 2010
	✗	H^+^	*S. enterica*	Suzuki et al., 2019
Na^+^ conduction	✓	Na^+^	*V. alginolyticus*	Fukuoka et al., 2009
	(✓)	Na^+^	*B. subtilis*	Terahara et al., 2017

*Marks in parentheses denote indirect evidence for assembly, either via measurements of motor/swimming speed (Fung and Berg, 1995; Zhou et al., 1998; Kojima and Blair, 2001; Sowa et al., 2005, 2014), wherein it cannot be ruled out that the stators are assembled but somehow inactive, or via a structural switch of the stator complex that suggests peptidoglycan (PG) binding (Terahara et al., 2017)*.

Studies that have found both motor speed and single stator speed to be linearly dependent upon IMF seem to make sense only under the assumption that the number of assembled stators is constant. Thus, the fact that stator assembly depends, in some yet-to-be-determined manner, on IMF complicates the interpretation of the linear dependence of speed on IMF. There exists an unresolved contradiction, as a linear relationship between IMF and speed should not be observed if both stator number and speed per stator increase simultaneously. It may prove that the relationship between stator number and IMF is sub-linear, and that stator disassembly mostly occurs only at very low IMF values. Future studies of stator dynamics as a function of IMF will be key to understanding how these pieces fit together.

## Ion Specificity

In most bacteria, stators are powered by a single species of cation, mostly commonly protons or sodium ions. Typically, MotAB-type stators are H^+^-coupled and PomAB and MotPS-type stators are Na^+^-coupled. Yet some bacteria are capable of coupling multiple types of cations. One way in which this is accomplished is via multiple sets of genes that encode multiple types of stator complexes. For example, *B. subtilis, V. alginolyticus*, and *S. oneidensis* all encode both an H^+^-driven MotAB stator and an Na^+^-driven MotPS or PomAB stator (Atsumi et al., 1992; Asai et al., 2000; Ito et al., 2005; Paulick et al., 2009). In *V. alginolyticus*, PomAB stators are constitutively expressed and power the polar flagellar motor, whereas MotAB stators are expressed only under certain environmental conditions and exclusively power lateral flagella motors (Belas et al., 1986; McCarter et al., 1988; Atsumi et al., 1992; Kawagishi et al., 1995). In *B. subtilis* and *S. oneidensis*, both types of stators are constitutive. They incorporate into the same motor, working in unison, though their affinity to the motor depends upon the chemical and physical conditions of the environment (Ito et al., 2005; Paulick et al., 2009). A systematic survey of bacterial genomic data has shown that at least 65 species of bacteria possess two or more putative stators (Thormann and Paulick, 2010). Other species couple multiple cations via a single stator complex. For example, the MotAB stator of *Bacillus clausi* can couple either H^+^ or Na^+^ (Terahara et al., 2008), and MotPS in *Bacillus alcalophilus* couples Na^+^, K^+^, or Rb^+^ (Terahara et al., 2012). While it was reported that MotAB in *Paenibacilus* sp. was unique in its ability to couple divalent cations Mg^2+^ and Ca^2+^ (Imazawa et al., 2016), recent results suggest that this stator is actually powered by monovalent ions, likely protons (Onoe et al., 2020). Regardless of the coupling ion, a universally conserved aspartate residue at the N-terminal side of the TM region of MotB (D32 in *E. coli*) is believed to serve as the site of ion binding (Zhou et al., 1998).

Many hybrid stators have been created in order to probe the question of what in the stator determines ion specificity. Early work demonstrated that the combination of MotA (*R. Sphaeroidis*) and PomB (*V. alginolyticus*) produced a Na^+^-driven stator in *V. alginolyticus*. This hybrid required MotX and MotY proteins in order to function, components which are necessary for PomAB incorporation in *V. alginolyticus* (Terashima et al., 2013). This work ruled out the A subunit as the ion-decisive component (Asai et al., 1999). In *B. subtilis*, hybrid stators MotAS and MotPB were shown to be Na^+^ and H^+^-coupled, respectively, suggesting that the B/S subunit is the dominant determinant of ion-selectivity (Ito et al., 2005). However, it was observed that MotA and MotP subunits confer an H^+^ or Na^+^ responsiveness, respectively (Ito et al., 2005). This suggests that either the A/P subunit plays a secondary role in ion-specificity, or, given the evidence presented above for stator sensing of IMF, it may be the case that the A/P subunit is a determining factor of H^+^ and Na^+^-dependent stator assembly. It was also recently proposed that the P subunit is critical for K^+^ ion selectivity in *B. alcalophilus* and *Bacillus trypoxylicola* (Naganawa and Ito, 2020).

To explore which portion of the B subunit determines ion specificity, a chimeric subunit, termed MomB, was constructed from the N-terminus portion of MotB (*R. Sphaeroidis*) and the C-terminus portion of PomB (*V. alginolyticus*). MomB and PomA produced an Na^+^ stator in *V. alginolyticus*, and interestingly, the position of the MotB/PomB junction within the conserved TM region affected the Na^+^/Li^+^ specificity. It was thus hypothesized that the periplasmic region of PomB proximal to the inner membrane plays a role in ion specificity, potentially by changing the size of the TM pore (Asai et al., 2000). Certain MomB constructs also produced a Na^+^-driven stator with MotA in *V. alginolyticus*, albeit its function was dependent upon the presence of MotX and MotY (Asai et al., 2000, 2003). The reverse construction, the N terminus of PomB combined with the C terminus of MotB, named PotB, functioned with PomA as a Na^+^-powered stator in *V. alginolyticus* or *E. coli*, regardless of presence of MotXY (Asai et al., 2003). Thus, while the periplasmic portion of PomB is not necessary for Na^+^ coupling in the PomAB stator, it is sufficient to convert the MotAB stator from H^+^- to Na^+^-coupling.

Mutations near the surface of the conserved TM segment of the B subunit can cause the dual-ion coupling stator of *B. clausi* to prefer either H^+^ or Na^+^, and mutations in the same region can confer a dual-ion coupling capacity to *B. subtilis* stators (Terahara et al., 2008). A single mutation in the TM region of MotS of *B. alcalophilus* changed it from multi-ion coupling to single ion coupling (Terahara et al., 2012). Sequence alignment revealed that H^+^-coupled MotB stators from many bacteria contain a conserved valine in the middle of the TM segment (residue 43 in *E. coli*), whereas Na^+^-coupled PomB and MotS stators contain a conserved leucine at this position, and the Na^+^/K^+^/RB^+^-coupled MotS of *B. alcalophilus* contains a methionine (Terahara et al., 2012). Steered molecular dynamic simulations of an atomic model of MotAB constructed based on disulfide cross-linking and tryptophan scanning mutations showed that the size of the ion channel at its narrowest point was sensitive to mutations at this TM residue, suggesting size exclusion as the mechanism for ion-selectivity (Nishihara and Kitao, 2015).

**Figure 3:**
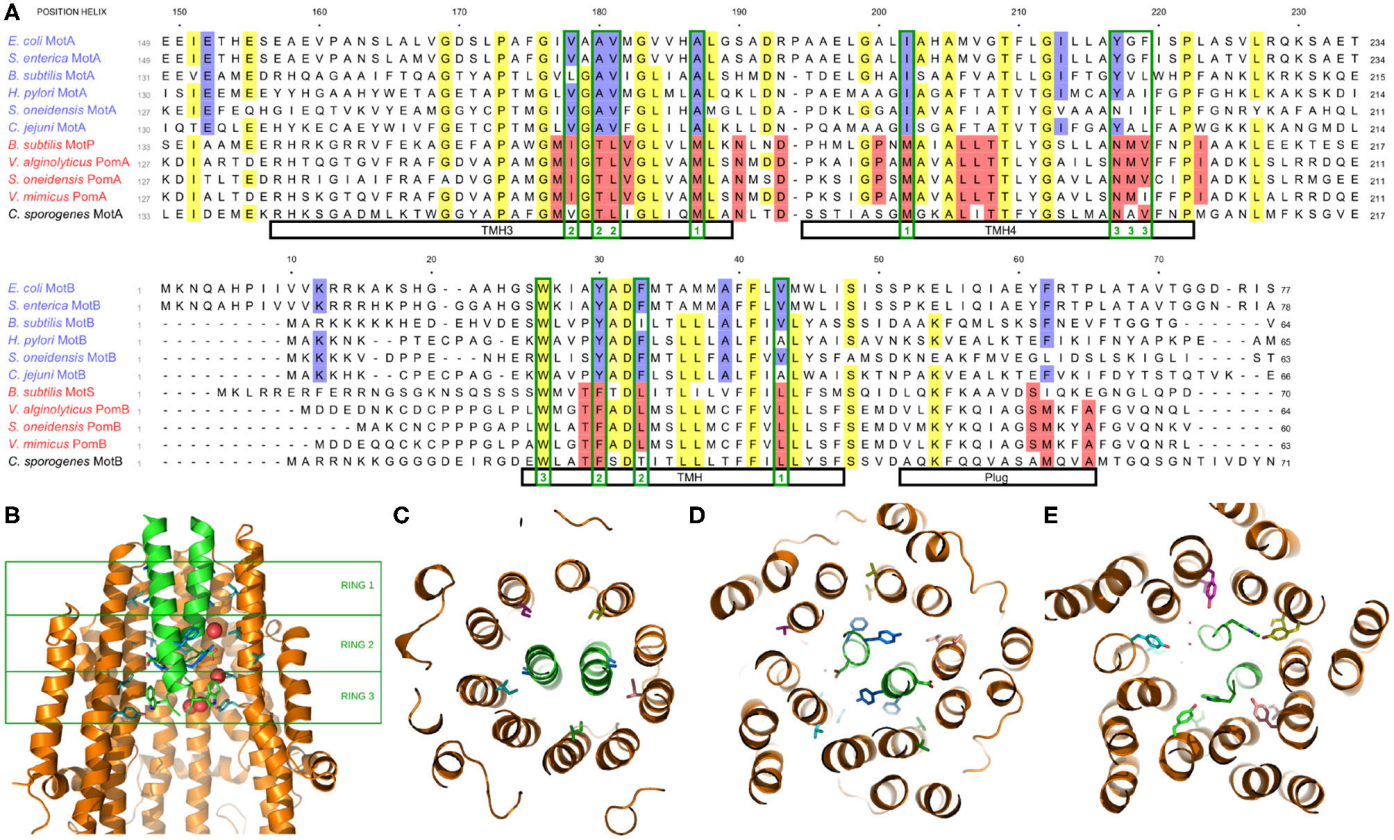
**(A)** Multiple species sequence alignment of the A and B stator subunits. Highlighting denotes >70% conservation across either all species (yellow) or differentially across only the H^+^ or Na^+^ stators (blue and red, respectively; the coupling ion of *C. sporogenes* remains unclear). Residues mentioned in the text which map to one of the three rings of differentially conserved residues are outlined in green with the corresponding ring noted at the bottom. **(B)** Side view of the stator, showing the two MotB and three of the five MotA (side chains shown for MotB and two MotA units). The three rings of differentially conserved residues are marked by the green boxes. **(C)** Bottom view (looking from the cytoplasm) of Ring 1. **(D)** Bottom view of Ring 2. **(E)** Bottom view of Ring 3.

Recently, the structure of the stator has been determined via cryoelectron microscopy (Deme et al., 2020; Santiveri et al., 2020). In Figure 3A, we show portions of a multiple sequence alignment, initially performed with the Clustal Omega alignment program (Madeira et al., 2019), then refined and edited using ViTO (Catherinot and Labesse, 2004), taking into account the published structures (PDB: 6YKP, 3S0Y). These alignments highlight the differentially conserved residues within the transmembrane helix (TMH) of MotB, but they also identify multiple differentially conserved residues in TMH3 and TMH4 of Mot/PomA that map to the A/B interface, which is very compact and largely hydrophobic in its most central part (Deme et al., 2020; Santiveri et al., 2020). The differentially conserved residues generally localize to three rings, as shown in Figure 3B, with no tight connections. Near the periplasmic interface, residue EcMotB_V43_ faces residues EcMotA_I202_ and EcMotA_A187_ within TMH4 and TMH3, respectively (Figure 3C). While this ring is fully buried and corresponds to hydrophobic substitutions, differences between the H^+^ and Na^+^ stators here may yield an overall change of size or flexibility of the region, as previously hypothesized (Nishihara and Kitao, 2015). Further into the channel, residues MotB_Y30_ and MotB_F33_ face residues 178–181 of MotA TMH3, and with more accessibility, this presents another region that may control ion specificity (Figure 3D). Substitutions at that ring will impact a network of interactions involving hydrogen bonds to the essential aspartate MotB_D32_. Indeed, an imperfect correlation between MotB residue 30 and ion specificity was previously noted (Ishida et al., 2019; Islam et al., 2020), and selection may prove to depend partly upon the facing TMH3 region. Finally, residues 217–219 of MotA TMH4 face the conserved MotB_W26_ residue (Figure 3), which forms an H-bond with MotA_Y217_ in H^+^ motors. In sodium MotP/PomA stators, a smaller and more flexible asparagine is observed, instead. As a MotB_W26A_ mutation abolishes motility (Deme et al., 2020), this contact is important, though we see no obvious mechanism of ion selection in this open region.

## Spatiotemporal Variations of IMF at the Cell and Population Level

The electrochemical gradient across the bacterial cell membrane is central not only for motility, but for other physiological processes from ATP synthesis (Mitchell, 1961; Maloney et al., 1974) to cell division (Strahl and Hamoen, 2010; Chimerel et al., 2012). For this reason, it was traditionally considered to be homeostatic. However, recent work has shown that the IMF can be dynamic, is integrally tied to the cell's physiological state, and plays a role in information signaling. IMF-mediated intra- and intercellular electrical signalings have recently been shown to regulate physiological processes such as mechanosensation and metabolic coordination, sometimes over long distances, either among or between communities (Liu et al., 2015; Bruni et al., 2017). We discuss here a few examples where spatiotemporal variation of the IMF has been observed, both at the single cell and population level.

As a pioneering work in the burgeoning field of bacterial electrophysiology, Kralj et al. (2011) developed a voltage-sensitive fluorescent membrane protein (PROPS) to probe voltage dynamics on the single cell level. They observed that, while cells show great heterogeneity in behavior, many cells displayed quasi-periodic fluctuations in their membrane voltage. Predictably, these transient depolarizations correlated to transient decreases in motor speed. The physiological role of electrical spiking was unclear at the time; as the intensity and frequency were affected by the power of the imaging laser, the authors hypothesized that such fluctuations may be a signature of a bacterial stress response. When PROPS was combined with a genetically encoded calcium sensor, it was observed that transient membrane depolarizations induced transient influxes of calcium, reminiscent of neuronal action potentials (Bruni et al., 2017). Evidence that such a phenomenon is a signature of signaling came from the observation that transient voltage-induced Ca^+^ influxes increased upon mechanical stimulation, and that such stimulation led to changes in protein concentrations at the level of individual cells (Bruni et al., 2017).

IMF dynamics have recently been shown to be important for surface-attached communities of bacteria, called biofilms. At the population level, a multi-electrode array system has shown that the intensity of electrical activity correlates with biofilm formation (Masi et al., 2015). With single cell level resolution, it has been shown that biofilms produce spatially propagating waves of oscillating membrane potential, and that these waves correlate with propagating waves of extracellular potassium. There is substantial evidence to support a model in which membrane depolarization is triggered by metabolic stress at the interior of a biofilm, which triggers the release of intracellular K^+^, which subsequently depolarizes neighboring cells. Thus, membrane potential is used to conduct long-range electrical signaling to coordinate the metabolic states within a growing biofilm, thereby increasing the fitness of the biofilm as a whole (Prindle et al., 2015). Signals transmitted by these propagating waves of potassium extend beyond the biofilm community. Spatially separated biofilms can capitalize upon this electrochemical signaling to coordinate a time-sharing behavior of resource consumption (Liu et al., 2017), and the swimming behavior of distant planktonic cells can be modified in a manner to recruit cells to join a biofilm. While the majority of this work has been done on *B. subtilis*, the mechanisms may well be generic among bacteria; electrical signaling from a *B. subtilis* biofilm attracts swimming cells from the evolutionary distant *P. aeruginosa* (Humphries et al., 2017).

There appears to be great cell-to-cell heterogeneity in both the steady-state and dynamic behavior of cellular IMF (Kralj et al., 2011). Recent evidence has shown that both the steady-state membrane voltage and the dynamic behavior in response to electrical stimulation is dependent upon a cell's proliferative capacity (Stratford et al., 2019). Additionally, electrical polarization of the cell plays a role in successful spore formation (Sirec et al., 2019). While it may seem that such cell-to-cell heterogeneity may interfere with long-distance electrical signaling, a model based upon percolation theory proposes that the number of cells capable of transmitting the electrical signal is consistent with a predicted phase transition. Thus, a dense biofilm composed of a heterogeneous mixture of signaling and non-signaling cells manages to propagate signals over long distances while minimizing the cost at the single-cell level (Larkin et al., 2018).

## Single Cell IMF Measurements

While the developing field of bacterial electrophysiology takes its inspiration from the advanced field of eukaryotic electrophysiology, the reduced bacterial cell size and the complexity of the membrane present novel challenges. Techniques employed in the study of eukaryotic cells, such as microelectrode and patch clamp approaches, so far remain impractical, lack single-cell resolution (Masi et al., 2015), or come at the expense of great physical perturbation (Martinac et al., 2008). While bacterial bioenergetics has historically been investigated via bulk population measurements (Kashket, 1985), emerging techniques are enabling measurements of both the electric and chemical components of the IMF at the single bacterial cell level.

Measurements of ΔΨ are most commonly made with Nernstian sensors (Lo et al., 2006, 2007; Kralj et al., 2011; Prindle et al., 2015; Sirec et al., 2019; Stratford et al., 2019; Mancini et al., 2020), small fluorescent molecules that diffuse across the membrane in accordance with the Nernst equation. Fluorescent measurements of the ratio of the extracellular to intracellular concentration at equilibrium reports upon ΔΨ. However, their permeability to Gram-negative bacteria is low, yielding a slow dynamic response (~ minutes), and so they are not suitable to report upon rapid fluctuations in ΔΨ. Additionally, while high concentrations are desired for improved signal-to-noise ratio, increasing concentrations of Nernstian dyes actually perturb membrane voltage (Sirec et al., 2019; Mancini et al., 2020). Thus, their application requires careful calibration (Mancini et al., 2020). As mentioned above, an alternative to probe ΔΨ is a genetically encoded voltage sensor called PROPS, engineered from green-absorbing proteorhodopsin (Kralj et al., 2011; Bruni et al., 2017). PROPS provides a significantly faster temporal response (~ 10 ms; Kralj et al., 2011), without the need for a dye-loading step, though at the cost of lower signal-to-noise ratio.

Measurements of the chemical component of the IMF require a reporter for the intracellular concentration of the coupling ion. Intracellular pH was first quantified on the single cell level using membrane-permeable pH-dependent fluorescent dyes (Siegumfeldt et al., 2000; Rasmussen et al., 2008; Kurre et al., 2013; Chao et al., 2017). A more recent approach, which obviates the need for a dye loading step and its potential invasive effects (Han and Burgess, 2010), uses genetically encoded fluorescent proteins, such as pH-sensitive derivatives of GFP (Miesenböck et al., 1998; Martinez et al., 2012; Kurre et al., 2013; Morimoto et al., 2016; Rupprecht et al., 2017; Arce-Rodríguez et al., 2019). Aside from excellent temporal (<1 s) and pH (<0.1) resolution (Kralj et al., 2011), a fusion between pHluorin and FliG of the BFM has shown local pH measurements with sub-cellular spatial resolution (Morimoto et al., 2016). PHluorin can also be combined with PROPS for simultaneous single cell measurements of ΔpH and membrane potential (Kralj et al., 2011; Bruni et al., 2017). Quantitative single cell measurements of intracellular sodium have been demonstrated using membrane-permeable fluorescent Na^+^ dyes and applied to studies of the hybrid Na^+^-driven motor of *E. coli* (Lo et al., 2006, 2007; Minamino et al., 2016). Relative measurements of cytoplasmic Ca^2+^ have been demonstrated in *E. coli* via a genetically encoded fluorescent Ca^2+^ sensor (Bruni et al., 2017).

The fact that the speed of the BFM is proportional to IMF provides an intriguing opportunity to exploit it as a measuring device for the IMF of the coupling ion. Recently, it has been shown that the BFM of *E. coli* can measure dynamic changes in the PMF during transient exposures to an ionophore as well as dynamic light-induced photodamage to the cell membrane (Krasnopeeva et al., 2019). As the temporal resolution of such measurements are theoretically limited only by the acquisition rate and the relaxation time of the particle attached to the flagellar filament (~ ms), the BFM presents as an exciting future tool to quantitatively explore rapid electrophysiological dynamics on the single cell level. However, as highlighted above, such measurements will be affected by stochastic stator dynamics, as well as other factors, such as second messenger-mediated binding of cytoplasmic protein YcgR to the rotor, which slows motor speed in *E. coli* (Boehm et al., 2010; Fang and Gomelsky, 2010; Paul et al., 2010).

## Perspectives

In species investigated thus far, the speed of the BFM is linear with IMF. It remains to be fully elucidated when and to what extent stator assembly depends on IMF, as well as the underlying mechanism of this electrical or chemical sensing. Future work will also need to decode how the linearity between speed and IMF arises if both single stator speed and stator assembly are proportional to IMF. Such details will contribute to models of the still elusive mechanism of torque generation, which will be greatly aided by recently solved structures of the stator complex (Deme et al., 2020; Santiveri et al., 2020). These structures will also open the way for future simulations to shed light on ion specificity and ion-dependent assembly.

With recent advances in single-cell electrophysiology techniques, the field is poised to learn a great deal about IMF dynamics and its intrinsic relation to diverse physiological processes, on scales from a single motor complex up to an entire bacterial colony. One of the best tools to investigate IMF dynamics may, ironically, be the motor that consumes it; yet, our understanding of the IMF and its rate of consumption by the BFM remains incomplete.

## Author Contributions

AB-B, FP, GL, and AN researched and wrote this review. All authors contributed to the article and approved the submitted version.

## Conflict of Interest

The authors declare that the research was conducted in the absence of any commercial or financial relationships that could be construed as a potential conflict of interest.
